# Approaches to Extracting Bioactive Compounds from Bark of Various Plants: A Brief Review

**DOI:** 10.3390/plants14182929

**Published:** 2025-09-20

**Authors:** Adrian Nisca, Corneliu Tanase

**Affiliations:** 1 Doctoral School of Medicine and Pharmacy, “George Emil Palade” University of Medicine, Pharmacy, Sciences and Technology of Târgu Mures, 38 Gheorghe Marinescu Street, 540139 Târgu Mures, Romania; 2 Department of Pharmaceutical Botany, Faculty of Pharmacy, “George Emil Palade” University of Medicine, Pharmacy, Sciences and Technology of Târgu Mures, 38 Gheorghe Marinescu Street, 540139 Târgu Mures, Romania; 3 Research Centre of Medicinal and Aromatic Plants, “George Emil Palade” University of Medicine, Pharmacy, Sciences and Technology of Târgu Mureș, 38 Gheorghe Marinescu Street, 540139 Târgu Mureș, Romania

**Keywords:** bark, bioactive compounds, extraction methods, phytochemicals, green methods

## Abstract

In recent years, focus has been directed toward studying lignocellulosic matter, such as forestry by-products, due to their high therapeutic potential offered by various bioactive compounds, mainly phenolic compounds. To obtain extracts rich in these phytochemicals, suitable extraction methods must be employed, and a thorough understanding of these methods is necessary. This work concentrates on describing both classical and modern extraction techniques, highlighting their mechanisms as well as their key advantages and disadvantages. It was observed that a wide variety of extraction methods are currently used for bark, emphasizing the importance of method optimization to achieve higher yields of phytochemicals valuable for their biological activities.

## 1. Introduction

### 1.1. Study Background

Plant barks are increasingly valued as sources of bioactive compounds due to their rich phytochemical content, including various phenolic compounds, flavonoids, tannins, and other secondary metabolites. Using trunk barks as a source of bioactive compounds supports the sustainable utilization of forest resources. Trunk barks are often by-products of the timber industry, and their valorization can help reduce waste and promote a circular economy [1]. Moreover, other barks such as root, stem, and cone barks may also be used to obtain extracts rich in phytochemicals with bioactive properties [2,3,4].

These bioactive compounds are renowned for their numerous health benefits and potential applications in various industries, including pharmaceuticals, nutraceuticals, and cosmetics. Barks from several tree species, including *Quercus* (oak) [5,6,7], *Salix* (willow) [8,9], and *Pinus* (pine) [10,11], contain high levels of phenolic compounds that display notable antioxidant, antimicrobial, and anti-inflammatory activities. The antioxidant capacity of bark extracts comes from their phenolic content, which can neutralize free radicals and lessen oxidative stress. For example, extracts from *Salix alba* L. have demonstrated potent antioxidant properties, making them suitable for health and cosmetic products [12]. Bark extracts from different plant species have shown significant antimicrobial activity against a variety of pathogens [4,13,14,15,16,17] and also inhibit key enzymes involved in various metabolic pathways [18,19,20]. Studies on different animal models have shown the antidiabetic properties of *Acacia mearnsii* De Wild., *Annickia polycarpa* DC., and *Callicarpa arborea* Roxb. Bark extracts, through various mechanisms such as protection against diabetes complications through their antioxidant properties, regeneration of pancreatic beta cells, AMPK activation, and DPP-4 inhibition [21,22,23]. Pine bark extracts have shown significant effects in humans, including lowering systolic and diastolic blood pressures, blood sugar levels, and LDL cholesterol, indicating potential use in the management and prevention of cardiovascular disorders [24]. *Terminalia arjuna* Roxb. Ex DC. bark indicated cardioprotective effects by increasing coronary blood flow, and thus protecting the myocardium against ischemic events [25]. In vitro studies have also shown that phytochemicals present in bark extracts may be effective against various cancer cell lines such as breast, cervical, colon, and pancreatic [26].

**Figure 1:**
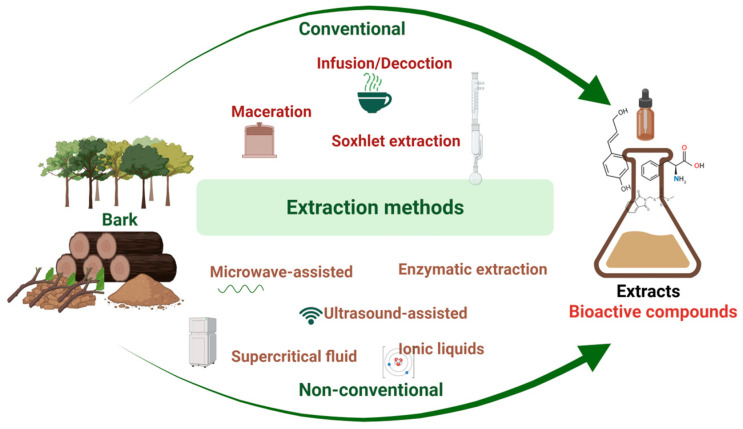
A schematic representation of the used extraction methods of bioactive compounds from bark.

The extraction process is crucial for obtaining bioactive compounds from bark extracts, as it directly impacts the yield, composition, and biological activity of the final extracts. Different extraction methods (such as maceration, ultrasound-assisted extraction (UAE), microwave-assisted extraction (MAE), and supercritical fluid extraction) can significantly influence the yield of phenolic compounds, tannins, and other bioactive substances (Figure 1) [13,27,28,29]. The choice of solvent also plays a vital role in extraction efficiency and the types of compounds that can be extracted. Polar solvents, such as methanol and ethanol, are often more effective at removing phenolic compounds, which are typically polar [30,31]. Additionally, extraction processes need to be optimized considering parameters like temperature, time, and solid-to-solvent ratio to maximize the extraction of bioactive compounds [27,32,33,34,35]. Efficient extraction methods can also reduce the co-extraction of unwanted compounds that may interfere with the desired biological effects. For example, deep eutectic solvents have been shown to selectively extract phenolic compounds while minimizing the presence of undesirable substances [36]. Lastly, the sustainability and environmental impact of extraction methods should be taken into consideration. The trend toward using green extraction techniques, such as UAE and MAE, reflects a growing awareness of ecological sustainability. These methods typically require less solvent and energy, making them more eco-friendly while still delivering high extraction efficiency [37,38,39,40,41].

### 1.2. Study Objectives

The review aims to examine extraction techniques for bioactive compounds from various plant barks, highlighting their potential health benefits and uses. It also discusses how these advanced methods can enhance the yield and quality of extracts, with a focus on recovering phenolic compounds, tannins, and other bioactive substances. Additionally, it emphasizes the importance of optimizing extraction parameters, such as solvent type and extraction time, to maximize the phytochemical content and biological activities of the extracts. Characterizing these extracts is crucial for understanding their chemical composition and potential therapeutic properties, which can lead to their application in pharmaceuticals, nutraceuticals, and cosmetics.

Overall, the review emphasizes the importance of a comprehensive approach to extracting bioactive compounds from plant barks, thereby promoting their valorization and utilization in various industries. This emphasis on extraction methods is crucial for advancing research in the field of natural products and their health benefits. 

## 2. Methodology

### 2.1. Botanical Terminology

All botanical names were verified for accuracy using the International Plant Names Index (IPNI) (https://www.ipni.org/, accessed on 16 August 2025) and the World Flora Online (WFO) Plant List (https://wfoplantlist.org/, accessed on 16 August 2025).

### 2.2. Data Collection

Searches were conducted for open-access articles published in journals indexed in international academic databases (PubMed, Springer, Scopus, ScienceDirect) over the past 13 years and were reviewed. Keywords such as “bark,” “phenolic compounds,” “extraction,” and “bioactive” were used in database searches.

## 3. General Overview of Bark Extraction

### 3.1. Chemical Composition of Plant Barks

Bark extracts contain a variety of primary and secondary metabolites, which contribute to their biological effects and potential health benefits. Here is an overview of some key metabolites commonly found in bark:

- Phenolic Compounds:
  - Phenolic Acids: Often found in bark, these include gallic acid, ellagic acid, and caffeic acid. They are recognized for their antioxidant effects and other bioactive properties such as antimicrobial and anti-inflammatory activities [31,42,43].
  - Flavonoids: This group includes compounds like catechin, quercetin, and taxifolin. Flavonoids are known for their antioxidant, anti-inflammatory, and antimicrobial properties [14,31,44].
  - Tannins: These are polyphenolic compounds that can be divided into hydrolyzable and condensed tannins (proanthocyanidins). Tannins are recognized for their astringent properties and potential health benefits, including antimicrobial and antioxidant effects [19,31,33].
  - Lignans: These are a group of phytoestrogens that can be found in bark and are known for their antioxidant and potential anticancer properties [31,32,42,45].
  - Coumarins: A class of organic compounds characterized by a benzopyrone structure, which consists of a benzene ring fused to a pyrone ring. They are widely found in the plant kingdom and are known for their diverse biological activities, including anticoagulant, anti-inflammatory, antimicrobial, and antioxidant effects. Coumarins can be found in various parts of plants, including leaves, fruits, and especially in the bark of specific tree species [14,45].
- Saponins: A class of glycosides defined by their surfactant properties, which come from their structure that includes a hydrophobic aglycone (sapogenin) attached to one or more hydrophilic sugar groups. These compounds are common in the plant kingdom and are known for their wide range of biological activities, including antifungal, antibacterial, antiviral, and anti-inflammatory effects. Saponins also exhibit potential health benefits, including the ability to lower cholesterol and enhance the immune system [46,47].
- Terpenoids: A class of organic compounds derived from five-carbon isoprene units. They are known for their strong aromatic properties and are commonly found in essential oils, resins, and various plant extracts. Terpenoids play crucial roles in plant defense mechanisms, attract pollinators, and protect against herbivores and pathogens. They exhibit a wide range of biological activities, including antimicrobial, anti-inflammatory, antioxidant, and anticancer effects [11,38,48].

### 3.2. Importance of Bark as a Source of Extracts

Bark is increasingly recognized as a valuable source of bioactive compounds, often showing significant antioxidant, antimicrobial, and anti-inflammatory properties. Extracting bioactive compounds from bark can produce higher concentrations of certain phytochemicals than other plant parts, such as leaves, fruits, and seeds [32,49]. For example, in the case of *Zanthoxylum armatum* DC., seeds exhibited lower antioxidant properties compared to bark extracts, as indicated by IC50 values, which suggest reduced efficiency [50]. Additionally, some studies have reported that specific compounds, like flavonoids and tannins, are more abundant in bark extracts. For instance, when comparing leaf and bark extracts from *Populus* species, bark extracts were found to contain higher amounts of bioactive compounds [44]. Furthermore, the bark of various *Quercus* species has been shown to contain higher total phenolic content than leaves, suggesting greater potential for bioactivity [1].

The composition of extracts can vary significantly between bark and other plant parts, leading to improvements in bioactivity, including antioxidant capacity [51,52], antimicrobial potential [1,15], and enzymatic inhibition [20].

Moreover, the pretreatment of the vegetal matrix, before the actual extraction, may also influence the final extract both qualitatively and quantitatively. For example, Moomin et al. have previously shown that bark extracts obtained from the bark of *Terminalia ivorensis* A.Chev., harvested in different seasons or storing for four years, significantly changed the phytochemical profile of the extracts [53]. Similarly, Halmemies et al. presented the effect of various storage periods (winter and summer) on Norway spruce bark, highlighting the faster degradation of phytochemicals during summer due to increased UV light exposure, higher temperatures, and more intense microbial activity [54]. Other factors, such as drying and pulverization of the vegetal matrix, might also enhance or decrease the extractive yields. Higher drying temperatures (above 60 °C) and longer drying times (more than 10 h) may cause certain phytochemicals, such as condensed tannins, to degrade quickly [55]. In contrast, a higher degree of pulverization of the bark may increase the yields of bioactive compounds [56], but only to a certain extent as finer powder particles tend to agglomerate, resulting in a lower penetrability of the solvent and thus to lower yields [57].

### 3.3. Extraction Principles and Challenges

Extraction principles and challenges are vital considerations in phytochemistry, especially when isolating bioactive compounds from plant materials like bark. To achieve optimal yield, factors such as the solvent, solvent-to-solid ratio, extraction techniques, temperature, time, pH, and ionic strength must be carefully adjusted based on the type of phytochemicals to be extracted and the phytochemical matrix used. The choice of solvent is essential because it influences the solubility of the target compounds. Polar solvents (e.g., water, methanol) are typically used to extract polar compounds like phenolics, while non-polar solvents (e.g., hexane, chloroform) are used for lipophilic compounds [10]. Often, solvent mixtures are employed to enhance extraction yields from plant materials [53], with combinations such as ethanol–water and methanol–water used to obtain extracts rich in phenolic compounds, including flavonoids and tannins [8,43,58,59]. Solvents like carbon dioxide may also be used for supercritical fluid extraction alone [48], and some studies suggest that combining ethanol with carbon dioxide can be more effective for extracting higher amounts of polar bioactive compounds like phenolics [15,38].

Besides the importance of solvent choice, the solid-to-solvent ratio can also significantly influence extraction efficiency. Therefore, optimizing this parameter is essential to maximize yields [36]. For example, when extracting phenolic compounds, a specific solid-to-solvent ratio is often established to enhance contact between the solvent and plant material, thereby improving the extraction yield [48,60]. A higher ratio might result in insufficient solvent to dissolve the target compounds, leading to lower yields. Conversely, a very low ratio can cause solvent saturation, which can also reduce extraction efficiency [35,61].

Temperature and time are also critical parameters of the extraction process. The extraction temperature influences the solubility of the compounds being extracted. Higher temperatures can increase extraction efficiency by enhancing the solubility of polar compounds, such as phenolics and flavonoids, which are commonly found in bark [55,62,63]. However, excessively high temperatures may also cause the degradation of sensitive compounds, so a balance must be maintained [33,55]. The duration also plays a key role in the extraction process, as sufficient time is required for the extraction to occur; however, an excessively long duration can negatively impact the quality of bioactive compounds [13,64,65,66].

Ionic strength and pH are key factors for the optimal extraction and preservation of bioactive compounds from different barks. Ionic strength can impact the extraction process by influencing the interactions between the solvent and the solutes [67], while pH may affect the solubility and stability of the desired compounds [68,69].

Finally, the extraction technique used in the process may be one of the most critical aspects of extraction, if not the most important. Therefore, this part will be expanded upon in the next section of the review.

After describing the principles and general aspects of extraction methods, it is also essential to mention the challenges faced by those who attempt to perform these methods. Plant materials often contain a complex mixture of compounds, making it difficult to selectively extract the desired bioactive components without also extracting unwanted substances [41]. Achieving high yields of pure extracts can be challenging, as extraction methods may also remove impurities that can affect the bioactivity of the final product [28]. Moreover, there are increasing concerns regarding the environmental impact of the organic solvents used for extractions, leading to a growing demand for “green” extraction methods [70,71,72]. Some extraction techniques, such as supercritical fluid extraction (SFE), can be costly and may not be easily scalable for industrial applications [28]. Lastly, extracts intended for food, pharmaceutical, or cosmetic use must meet strict regulatory standards, which can complicate the extraction process and necessitate extensive testing [73].

In summary, although the principles of extraction are well-established, the challenges involved in optimizing extraction processes for specific bioactive compounds remain substantial. Ongoing research strives to develop more efficient, sustainable, and selective extraction methods to improve the recovery of valuable phytochemicals from plant materials.

## 4. Extraction Techniques for Bark Extracts

### 4.1. Water-Based Extraction Methods (Infusion/Decoction)

Water-based extraction methods, such as decoction and infusion, are commonly used to extract bioactive compounds from plant materials, including barks. These methods rely on water’s solvent properties to dissolve various phytochemicals, making them effective for obtaining extracts rich in beneficial compounds. Decoction involves boiling the plant material in water for a set period. Gatsou Djibersou et al. described preparing aqueous extracts from *Bixa orellana* L. bark using this method by boiling the bark powder in distilled water for 20 min, resulting in a yield of 4.68% [65]. Additionally, Tienaho et al. discuss the extraction of bioactive compounds from *Salix* spp. bark with hot water, suggesting that decoction may be advantageous for tougher plant materials [9]. In contrast, infusion is a gentler method, usually involving pouring boiling water over the plant material, and it is more suitable for compounds sensitive to high temperatures, such as flavonoids and volatile oils [59]. Articles describing infusion protocols for extracting bioactive compounds from the bark of *Allophylus abyssinicus* Radlk., *B. orellana*, *Juglans regia* L., and *Symphonia globulifera* L.f. [59,65,74] can be found in the literature. Furthermore, considering the cost and environmental benefits, research has been conducted on scaling these extraction techniques for industrial use, as well as on addressing the challenges of upscaling [73,75,76,77]. Efforts have also focused on improving water-based extraction by optimizing parameters [78] and comparing it to other solvent-based techniques [15], which generally show lower efficiency with water-based methods [74,76,77].

### 4.2. Maceration

The maceration process is a widely used extraction method to obtain bioactive compounds from plant materials. It involves soaking the plant material in a solvent to help release soluble compounds. For example, bark is first dried and ground to increase surface area, which improves extraction efficiency. The solvent is chosen based on the polarity of the compounds intended for extraction [16,39,55]. The ground material is then mixed with the solvent in a container, and the mixture is left to soak for a set period, ranging from several hours to days. During this time, the mixture is often stirred or agitated to improve contact between the solvent and the plant material, promoting the release of soluble compounds. Pap et al. used a soaking time of 60 min at either 40 °C or 50 °C, with constant magnetic agitation during the extraction of various plant materials, including Scots pine and Norway spruce barks [64]. Truong et al. employed a 24 h soaking period with different temperature protocols and continuous agitation to optimize the extraction yield from *Severinia buxifolia* Ten. bark and branches [47,62]. After soaking, the mixture is filtered to remove the solid plant material from the liquid extract, which can then be concentrated using techniques such as rotary evaporation to remove the solvents [18,19,79,80].

Maceration is a commonly used method for extracting bark compounds, with several benefits, drawbacks, and restrictions. Its primary advantage is that it is a simple and easy-to-implement technique that does not require specialized equipment, making it accessible to many users [45,62,79]. Additional benefits include its cost-effectiveness [20], ability to preserve heat-sensitive compounds [81], and the potential to yield high amounts of certain compounds, typically polar phytochemicals [62]. The main disadvantages and limitations include the lengthy time often required for optimal extraction [20], limited control over extraction conditions, as maceration generally lacks the precision found in more advanced methods [55], and lower yields compared to these methods [32,39].

Several articles highlight the biological activity of macerates derived from the bark of various plant species. Habibou et al. highlighted the antioxidant and anti-Shigella activities of *Detarium microcarpum* Guill. & Perr. root bark [82], Shahwar et al. evaluated the anti-inflammatory potential of extracts obtained from the stem bark of *Ziziphus jujuba* Mill. [83], and Kajszczak et al. investigated the inhibitory effects of *Viburnum opulus* L. bark on carbohydrate digestion, demonstrating its potential as an antidiabetic agent [68]. These findings suggest that maceration remains a viable extraction method for obtaining highly valuable phytochemical extracts.

### 4.3. Soxhlet Extraction

The Soxhlet extraction principle is a method used to extract compounds from solid materials, especially useful for obtaining lipophilic substances. The Soxhlet apparatus includes a round-bottom flask, a Soxhlet extractor, and a condenser. The solid sample is placed in a porous thimble, which is then positioned in a Soxhlet extractor [69,84]. A solvent is added to the flask and heated. As the solvent heats, it vaporizes and rises into the condenser, where it cools and condenses back into liquid. This liquid then drips into the thimble containing the solid sample [85]. The solvent extracts the target compounds from the solid. When the solvent level in the extractor reaches a certain height, it siphons back into the flask, carrying the dissolved compounds with it [50,84]. This cycle of vaporization, condensation, and siphoning continues for several hours or even days in some cases, allowing for the thorough extraction of compounds from the solid material [17,86,87]. Table 1 presents some Soxhlet extraction protocols described in the literature.

Some advantages of the Soxhlet method include the continuous recycling of the solvent, which leads to higher extraction yields compared to techniques that use a single batch of solvent [50,69], the flexibility of using both polar and non-polar solvents [44], minimal sample handling [17], and consistent results thanks to its standardized process [53].

Even considering these advantages, the Soxhlet method has become outdated mainly due to its long extraction times (hours or even days) and high energy consumption [40]. The high temperatures used can cause thermal degradation of compounds [53], and there is a risk of artifact formation from prolonged heat and solvent exposure [44]. Additionally, the method is not environmentally friendly due to the energy-intensive heating [94] and the large volumes of organic solvents required [84,87].

### 4.4. Microwave-Assisted Extraction (MAE)

Microwave-assisted extraction (MAE) is a technique that uses microwave energy to heat solvents in contact with a sample, thereby enhancing the extraction of bioactive compounds. The process involves microwave radiation interacting with polar molecules and ionic species in the solvent and plant matrix, resulting in rapid heating and enhanced mass transfer.

Polar solvents and compounds absorb microwave energy, causing molecular dipoles to rotate and ions to move, which generates heat rapidly within the sample matrix [48,70]. This direct interaction between microwave radiation and polar molecules results in heating rates 10–100 times faster than conventional methods, facilitating the breakdown of plant cell walls and enhancing extraction efficiency [34]. The disruption of the vegetal matrix’s cell wall is explained by the rapid increase in temperature and internal pressure from evaporating cell moisture, which exerts pressure on the cell wall [8], leading to better solvent penetration and increased extraction efficiency [60,95]. Table 2 presents some studies that employed an MAE protocol for extracting the bark of various plant species.

Microwave-assisted extraction is a widely used, efficient extraction method that is fast and energy-saving, enhancing yields and quality, especially for polar bioactive compounds [8,34,70]. However, it requires careful optimization to avoid thermal degradation [60,96] and may involve higher initial equipment costs for specialized reactors. Nonetheless, studies show that regular microwave ovens can be effectively used for extracting lignocellulosic matrices, thereby reducing costs [51,70]. These features make MAE a promising green extraction technique with broad potential in natural products extraction and valorization.

### 4.5. Ultrasound-Assisted Extraction (UAE)

Ultrasound-assisted extraction (UAE) mainly involves the collapse of cavitation bubbles formed in the solvent when exposed to ultrasound radiation (cavitation process), generating localized high temperatures and mechanical effects that disrupt cell walls in the plant material, enhancing solvent penetration and increasing the contact area between solid and liquid phases. This results in improved extraction efficiency of bioactive compounds [37,48]. It has been shown that the extraction process using this technique typically follows a first-order kinetic model, indicating a rapid initial increase in yield due to higher mass transfer efficiency, followed by a slower rise as equilibrium nears [66]. Numerous studies describe protocols used for extracting bioactive compounds from the barks of various species with UAE, as summarized in Table 3.

One of the main advantages of ultrasound-assisted extraction is its high efficiency, providing a higher yield with less solvent and energy use, and also being faster [66] compared to other extraction methods [37,102]. It can even be more effective than microwave-assisted extraction for certain compounds [52]. Another significant benefit is that the technique works well at moderate temperatures, making it suitable for plant matrices that contain heat-sensitive phenolics [48]. Additionally, it is a method compatible with green solvents, which are more environmentally friendly [11,36,37].

The main disadvantage of using the UAE is the need for careful optimization of every extraction parameter (i.e., extraction time, temperature, solvent, power) not to affect the quality of the compounds comprised in the extract [34,37,66].

### 4.6. Supercritical Fluid Extraction (SFE)

Supercritical fluid extraction (SFE) relies on the unique properties of supercritical fluids, most commonly supercritical carbon dioxide (SC-CO_2_), which display characteristics between gases and liquids. When CO_2_ exceeds its critical temperature and pressure (31.1 °C and 73.8 bar), it enters a supercritical state where it has gas-like diffusivity and liquid-like density. This combination enables SC-CO_2_ to penetrate solid matrices like a gas but dissolve compounds like a liquid, improving mass transfer and the solubility of target substances [106]. The extraction process is affected by factors such as pressure, temperature, flow rate, extraction time, and the use of co-solvents (e.g., ethanol) to alter polarity and enhance the solubility of more polar compounds [107,108,109]. After extraction, reducing pressure in a separator causes the supercritical fluid to revert to gas, losing its solvent ability and precipitating the extracted compounds for collection. The CO_2_ gas can then be recycled or vented [109]. Several articles detail experimental conditions for the SFE of bark from various species, as shown in Table 4.

The main advantages of using supercritical liquid extraction methods are primarily sustainability and environmental friendliness, as they utilize supercritical carbon dioxide, which can be recycled and reused. Additionally, the final product contains no residual solvents, which is highly advantageous for producing food-grade ingredients, nutraceuticals, and natural products for pharmaceutical and cosmetic applications [48]. Other benefits of this extraction method include the low temperatures used during extraction, which help prevent thermal degradation of certain phytochemicals [33], the ability to adjust the solvent properties of CO_2_ by modifying pressure and temperature [29], and the method’s applicability to various biomass and waste materials such as bark, supporting biorefinery concepts and the valorization of renewable resources [29,48].

Although SFE is a modern and environmentally friendly extraction method, it has some limitations, such as the need for careful optimization of process parameters, because improper settings can lead to suboptimal yields or selectivity, making the process complex and sometimes difficult to scale up [33,38]. Additionally, supercritical CO_2_ is a non-polar solvent, which limits its ability to extract polar compounds unless polar co-solvents like ethanol are added; this requirement increases process complexity and can affect the purity of the extracts [29,48].

### 4.7. Other Methods

#### 4.7.1. Ionic Liquids Extraction

The primary benefits of ionic liquids stem from their combined use with advanced extraction methods such as microwave-assisted extraction (MAE) and vacuum microwave-assisted extraction (VMAE) [67,112]. Ionic liquids quickly and efficiently absorb microwave energy, resulting in faster heating and disruption of plant cell walls [67]. Specifically, imidazolium-based ionic liquids can dissolve cellulose and break hydrogen bonds, which increases cell wall permeability and allows better solvent penetration [113]. Additionally, Strehmel et al. found that ionic liquid extraction can selectively extract certain compounds while leaving others behind, such as glycosides in ionic liquid extracts of *Betula pendula* Roth bark, thus reducing the need for extra processing steps [114].

Ionic liquid extraction is a green, efficient, and sustainable method that provides higher extraction yields, lower energy consumption, protection of sensitive compounds, and solvent recyclability, making it a better alternative to traditional organic solvent extraction methods for natural products [115].

The main disadvantages highlighted for this type of extraction is represented by the high costs imposed by the ionic liquids, thus limiting industrial scale-up, high viscosity in the case of certain ionic liquids such as imidazolium ionic liquids that may cause handling and mass-transfer issues, often requiring dilution or solvents to lower viscosity, depending on the anion and cation alkyl chain length of certain ionic liquids that enter the environment through wastewater, they may harm aquatic and terrestrial organisms and some difficulties isolating/purifying the dissolved compounds from ionic liquid solutions [113,114,116].

#### 4.7.2. Enzymatic Extraction

The general process of enzymatic extraction involves using specific enzymes to break down the complex structural parts of plant cell walls (such as polysaccharides including cellulose, hemicellulose, lignin, and pectin), which helps release and recover bioactive compounds like polyphenols. Enzymes such as cellulase, protease, α-amylase, pectinase, and others catalyze the hydrolysis of specific bonds within these polysaccharides and proteins. This enzymatic activity leads to the breakdown of the cell wall structure and the release of intracellular bioactive compounds, including phenolics and peptides, which are generally hard to extract [117]. The hydrolysis caused by enzymes also alters the polyphenol profiles by releasing bound phenolic compounds and making them more easily extractable. Using enzymes together (for example, α-amylase and protease) often produces a synergistic effect, resulting in a more complete breakdown of cell walls and higher yields of phenolics and antioxidants compared to using a single enzyme or conventional solvent extraction [117].

Furthermore, enzymatic pretreatment can break down cell wall polysaccharides, increasing substrate porosity and facilitating solvent penetration, which enhances the extraction efficiency of both polyphenols and non-polyphenolic compounds [118].

Generally, enzymatic hydrolysis is often combined with physical methods, such as ultrasound or microwave-assisted extractions, and optimized solvent systems to enhance the extraction efficiency of polyphenols and other bioactive compounds [117,118,119]. However, as any other method, there are some drawbacks of using this method such as the potential polyphenol degradation with prolonged incubation, the sensitivity of enzymes to pH/temperature/time (need for careful optimization, increasing experimental complexity and cost), the release of non-target compounds that complicate downstream purification and may affect product purity and longer overall processing time [117,118,119,120].

#### 4.7.3. Deep Eutectic Solvent Extraction

Deep eutectic solvent (DES) extraction works by using a tailor-made eutectic mixture (a hydrogen-bond acceptor, HBA, plus one or more hydrogen-bond donors, HBDs) that has a lowered melting point and solvent properties tuned to dissolve target biomolecules. DES are prepared by mixing appropriate HBA and HBD components (e.g., choline chloride and organic acids, glycols, urea, and amino acids) and gently heating or stirring until a homogeneous liquid forms; water is frequently present and acts as a co-component that adjusts viscosity, polarity, and mass transfer [70,121,122]. DES dissolve target compounds via extensive hydrogen-bonding networks and complementary polarity/solvatochromic interactions that can compete with and disrupt native intermolecular bonds in the biomass (e.g., hydrogen bonds of polysaccharides or lignin–matrix interactions), increasing solute solubility and release [70,122,123].

Coupling DES with energy-assisted techniques (microwave or ultrasound) enhances extraction by rapid internal heating, cavitation, and mechanical disruption of cell walls, lowering residence time and reducing side reactions or degradation (microwave-DES for lignin [70] and ultrasound-DES for polyphenols and polysaccharides [36,122,124]). DES enables high selectivity and purity for polar biopolymers and phenolics, reduces volatility and toxicity compared to organic solvents, and, when combined with microwaves or ultrasound, results in much faster, lower-energy extraction [70,125]. In contrast, high viscosity can limit mass transfer, water content may strongly influence the performance of extraction and some DES compositions can cause hydrolysis or degradation of sensitive bioactive compounds [121,122].

### 4.8. Summary of Key Advantages and Drawbacks of Extraction Methods

Considering the data shown in the literature and highlighted in the current review, Table 5 comprises the main strong points of each extraction method described while also drawing attention towards certain drawbacks and challenges faced when using them, by analyzing extraction efficiency, technical complexity, feasibility of scale-up at industrial level and cost effectiveness (time and capital).

### 4.9. General Recommendations for the Selection of Extraction Methods

For the best selection of the extraction method and optimal yield, the characteristics of the phytochemicals to be extracted must be considered. If the compounds to be extracted are thermolabile, UAE might be the best option due to its rapid and low-temperature processing using a hydroethanolic mixture, while optimizing the solid–liquid ratio and extraction times [28,61]. MAE is an effective method for extracting phenolic compounds, utilizing minimal solvent and employing rapid heating. Optimization of microwave power and extraction time is a key factor for efficient extraction. The use of a vacuum during MAE is an essential addition when working with thermolabile compounds [13,112]. When the target compounds are lipophilic, SFE is the best option as it excels at extracting resins and volatile compounds. By adding small amounts of ethanol, the range of extractable phytochemicals is widened, increasing the capabilities of extracting semi-polar phenolics. Additionally, using multistage extraction conditions may enhance the selectivity of bioactive compounds [29,58].

Moreover, optimizing key physicochemical parameters is a critical step, regardless of the extraction method used. The use of hydroalcoholic mixtures may maximize the extraction capabilities for poorly water-soluble compounds while preserving matrix hydration; however, excessive ethanol may reduce solvent penetration [39,44]. Lower extraction temperatures must be used for thermolabile phytochemicals, as high temperatures may increase yields but may degrade sensitive phenolics [28,53]. Extraction time must be carefully adjusted, as longer macerations can increase yields but also raise the degradation risk. Therefore, it is recommended to use methods such as UAE and MAE, as these methods significantly reduce extraction times [61,69]. Solid-to-solvent ratios must be adjusted to avoid extract saturation, as this parameter represents a dominant factor for UAE [28,36]. The pH of extracts must also be carefully monitored, as water only extraction of tannin-rich bark may self-acidify and precipitate tannins. At the same time, the acidification of solvents may improve alkaloid extraction [44,93].

In Table 6, a comparison was performed regarding the different phytochemical profiles obtained from the same vegetal matrix by using various extraction methods, thus highlighting similarities and differences between yields and bioactive compounds extracted.

## 5. Conclusions

Across diverse bark matrices, extraction science is focusing on fast, selective, and more environmentally friendly workflows. Traditional methods (maceration, Soxhlet) remain useful for benchmarking and fractionation. Still, they are increasingly surpassed in yield, selectivity, process time, and energy use by ultrasound-assisted extraction (UAE), microwave-assisted extraction (MAE), and supercritical CO_2_ (SC-CO_2_). These methods are often used in cascades or combined with ionic solvents or enzymes to further enhance the results. Supercritical CO_2_ (SC-CO_2_) efficiently recovers lipophilic fractions and, after solvent extraction, enables higher recovery of polar metabolites. Meanwhile, microwave-assisted extraction (MAE) and ultrasound-assisted extraction (UAE) are specifically suited for extracting polar compounds, such as phenolics, often reducing extraction times and increasing yields without damaging heat-sensitive bioactive compounds. Additionally, combining these techniques with ionic or deep eutectic solvents and enzymes enhances their extraction capacity. However, to realize their full potential, thorough optimization of extraction parameters for each specific plant matrix is essential.

Given the current understanding of extraction methods, future research should focus on developing more specific, sequential, and solvent-smart techniques that are faster, cleaner, and more selective. These advances would enable the extraction of high-value ingredients for pharmaceuticals, nutraceuticals, and cosmetics while promoting the circular use of forestry residues.

## Figures and Tables

**Table 1:** Soxhlet extraction protocols.

Plant Species	Plant Material	Solvents	Time of Extraction	References
*Olea ferruginea* R.	Bark	n-Hexane, Chloroform, Methanol	-	[84]
*Z. armatum*	Fruit, Seed, Bark	Methanol	72 h	[50]
*Cassia fistula* L.	Stem bark, Small branches	Hexane, Chloroform, Ethyl-acetate, Ethanol	24 h	[88]
*Mangifera indica* L.	Leaves, Bark	Ethanol-water (8:2 *v*/*v*), Hexane	8 h	[69]
*Castanospermum australe* A. Cunn.	Leaves, Bark, Seeds	Petroleum ether, Benzene, Ethanol	-	[87]
*Acacia macrostachya* Rchb. ex DC.	Stem bark	Methanol	8 h	[17]
*Picea abies* L.	Bark	Ethanol-water (70% *v*/*v*)	6 h 34 min	[40]
*Rhamnus californica* Eschsch.	Leaves, Bark	Hexane, Dichlormethane, Methanol	24 h	[89]
*Umbellularia californica* Nutt.				
*Spondias dulcis* G.Forst.	Leaves, Stem bark	Ethyl acetate, Methanol, Water	6 h	[43]
*Spondias mombin* L.				
*Vateria indica* L.	Stem bark	Ethanol	12 h	[90]
*Azadirachta indica* A.Juss.	Stem bark	Methanol	6 h	[91]
*Populus* spp.	Bark	n-Hexane, Dichlormethane, Ethyl acetate, Methanol, Water	24 h	[44]
*T. ivorensis*	Bark	Petroleum ether, Chloroform, Ethyl acetate, Ethanol	118 min 125 min 110 min 115 min	[53]
*Pinus densiflora * Siebold & Zucc.	Bark	Ethanol, Methanol, Isopropanol, Acetonitrile, Acetone, Water	9 h	[10]
*Ulmus wallichiana* Planch.	Bark	Petroleum ether, Chloroform, Ethyl acetate, Methanol	-	[92]
*Phellodendron* spp.	Bark	Ethanol (70% *v*/*v*), HCl/Methanol (1:100 *v*/*v*), HCl/Ethanol (1:100 *v*/*v*), HCl/Water (1:100 *v*/*v*)	3 h	[93]
*Morus nigra* L.	Leaves, Bark	Ethanol (70% *v*/*v*)	7 days	[86]

**Table 2:** Microwave-assisted extraction protocols.

Plant Species	Plant Material	Solvent	Experimental Parameters	References
*Saraca asoca* Willd.	Bark	MeOH 50%, 70%, 90%	Material/solvent ratio = 1:10, 1:20, 1:30 Extraction time: 10, 20, 30 min Power: Fixed (not specified)	[60]
*Quercus rubra* L.	Bark	EtOH 70%, Water	Material/solvent ratio = 1:20 Extraction time: 30 min (aqueous extract); 18 min (ethanolic extract) Power: 850 W (aqueous extract); 650 W (ethanolic extract)	[1]
*Quercus cerris* L.	Bark	EtOH 70%, Water	Material/solvent ratio = 1:20 Extraction times: 10, 20, 30 min Power: 200, 500, 1000 W	[13]
*Z. jujuba*	Bark	MeOH	Material/solvent ratio = 1:50 Extraction time: 4 min Power: 100 W	[35]
*Fagus sylvatica L.*	Bark	EtOH 50%, 80%, Water	Material/solvent ratio = 1:10 Extraction time: 2, 3, 4 min Power: 300, 450, 600, 800 W	[51]
*Q. rubra*, *Q. cerris*, *Quercus robur* L., *Quercus petraea* Liebl., *Quercus dalechampii* Ten., *Quercus polycarpa* Schur	Bark	EtOH 80%	Material/solvent ratio = 1:250 Extraction time: from 30 s to 5 min Power: 700 W	[31]
*Pinus koraiensis* Siebold & Zucc.	Cone bark	EtOH 50%, Water	Material/solvent ratio = 1:10 Extraction time: 6 min (water extract); 4 min (ethanol extract) Power: 200 W	[4]
*Albizia myriophylla* Benth.	Bark	EtOH 60–100%	Material/solvent ratio = 1:20–1:40 Extraction time: 20–40 min Power: 400–900 W	[96]
*Ziziphus joazeiro* Mart.	Bark	Ethyl acetate	Material/solvent ratio = 1:100 Extraction time: 10–20 min Power: 300 W	[97]
*Populus tremula* L., *Pinus sylvestris* Thunb.	Bark	Water	Material/solvent ratio = 1:5 Extraction time = 5–30 min Power: not specified Temperature: 70–150 °C	[95]
*Larix decidua* Mill.	Bark	Choline chloride: Acetic acid (1:2)	Material/solvent ratio = 1:10 Extraction time: 30 min Power: 200 W	[70]
*Q. dalechampii*, *Quercus frainetto* Ten.	Bark	EtOH 70%, Water	Material/solvent ratio = 1:20 Extraction time: 18 min (ethanolic extract); 30 min (water extract) Power: 650 W (ethanolic extract); 850 W (water extract)	[30]
*P. sylvestris*, *Pinus nigra* Aiton	Bark	EtOH 50%	Material/solvent ratio = 1:10 Extraction time: 4 min Power: 300 W	[11]
*P. abies*	Bark	EtOH 50%	Material/solvent ratio = 1:10 Extraction time: 4 min Power: 300 W	[52]
*P. abies*	Bark	Water, EtOH 50%, EtOH100%	Material/solvent ratio = 1:12.5 Extraction time: 18 min Power: 200 W	[48]
*S. alba*	Bark	EtOH 70%, Water	Material/solvent ratio = 1:20 Extraction time: 18 min (ethanolic extract); 30 min (water extract) Power: 650 W (ethanolic extract); 850 W (water extract)	[8]
*Pinus brutia* Ten.	Bark	EtOH	Material/solvent ratio: 1:10 Extraction time: 10 min Power: 900 W	[34]
*Streblus asper* Lour.	Bark	EtOH	Material/solvent ratio = 1:10 Extraction time: 20 min Power: 350 W	[98]

**Table 3:** Ultrasound-assisted extraction protocols.

Plant Species	Plant Material	Solvent	Experimental Parameters	References
*P. abies*	Bark	EtOH 50% EtOH 70%	Material/solvent ratio = 1:10 Extraction time: 30, 45, 60 min Temperature: 40, 50, and 60 °C Frequency: 35 kHz	[37]
*S. alba*	Bark Leaves	MeOH 70%	Material/solvent ratio = 1:30 Extraction time: 30 min Temperature: 40 °C	[99]
	Bark	Water EtOH 50%	Material/solvent ratio = 1:40 Extraction time: 15 min Temperature: 70 °C Frequency: 40 kHz	[8]
*Q. rubra*	Bark	Water EtOH 50%	Material/solvent ratio = 1:40 Extraction time: 15 min Temperature: 70 °C	[1]
*Uncaria tomentosa* Willd. ex Schult.) DC.	Leaves Bark Stems Wood	MTBE:MeOH = 90:10 MeOH	Material/solvent ratio = 1:20 Extraction time: 30 min Temperature: 25 °C	[49]
*Z. jujuba*	Bark	MeOH	Material/solvent ratio = 1:50 Extraction time: 20, 30, 40, 50, 60 min Temperature: 25 °C Frequency: 35 kHz	[35]
*Semialarium mexicanum* Mennega	Root bark	Petroleum ether, EtOH, Water	Material/solvent ratio = 1:10 Extraction time: 30 min Temperature: 30 °C Frequency: 25 kHz	[100]
*Salix spp.*	Bark Leaves	Water	Material/solvent ratio = 1:100 Extraction time: 30 min Temperature: 25 °C Frequency: 40 kHz	[14]
*Q. dalechampii* *Q. frainetto*	Bark	EtOH 70% Water	Material/solvent ratio = 1:40 Extraction time: 15 min Temperature: 70 °C Frequency: 40 kHz	[30]
*P. sylvestris* *P. nigra*	Bark	EtOH 70%	Material/solvent ratio = 1:10 Extraction time: 30 min Temperature: 30 °C Frequency: 40 kHz	[11]
*Pouteria cambodiana* Baehni	Bark	EtOH 50% EtOH 60% EtOH 70%	Material/solvent ratio = 1:20 Extraction time: ND Temperature: 40, 50, 60 °C Frequency: 20 kHz	[46]
*Stryphnodendron adstringens* (Mart.) Coville	Bark	EtOH 65% EtOH 80% EtOH 95%	Material/solvent ratio = 1:250, 1:166.67, 1:125 Extraction time: 15, 30, 45 min Temperature: ND Frequency: 40 kHz	[61]
*P. abies*	Bark	EtOH 70%	Material/solvent ratio = ND Extraction time: 30 min Temperature: 65 °C Frequency: 40 kHz	[52]
	Bark	EtOH EtOH 50% Water	Material/solvent ratio = 1:12.5 Extraction time: 30 min Temperature: ND Power: 200 W	[48]
*Abies nephrolepis* Maxim.	Bark Leaves	EtOH 10–90%	Material/solvent ratio = 1:10–1:30 Extraction time: 30–50 min Temperature: 20–60 °C Frequency: 45, 80, 100 kHz	[66]
*Berberis vulgaris* L.	Root bark	Glycerol 10% Glycerol 50% Glycerol 90%	Material/solvent ratio = 1:50 Extraction time: ND Temperature: 20, 50, 80 °C Power: 144, 432, 720 W	[101]
*Pinus pinaster* Aiton	Needle Bark	Levulinic acid: formic acid = 70:30	Material/solvent ratio = 1:4–1:20 Extraction time: 1–2 h Temperature: 30–60 °C Frequency: 37 kHz, amplitude 20–80%	[36]
*Ilex rotunda* Thunb.	Bark	MeOH 30–100%	Material/solvent ratio = 1:20 Extraction time: 10–50 min Temperature: 20–80 °C Frequency: 40 kHz	[102]
*Cinchona* spp.	Bark	MeOH:NaOH 0.1 M = 49:1	Material/solvent ratio = 1:200 Extraction time: 20 min Temperature: ND	[103]
*P. brutia*	Bark	Water Ethyl acetate	Material/solvent ratio = 1:5 Extraction time: 20, 40, 60 min Temperature: 40, 70, 100 °C	[34]
*Frangula alnus* Mill.	Bark	MeOH 80%	Material/solvent ratio = 1:30 Extraction time: 15 min Temperature: ND	[104]
*Endopleura uchi* Cuatrec.	Bark	EtOH 50%	Material/solvent ratio = 1:100 Extraction time: 30 min Temperature: ND	[105]

**Table 4:** Supercritical fluid extraction protocols.

Plant Species	Plant Material	Co-Solvents	Experimental Parameters	References
*P. abies*	Bark	Ethanol	Pressure: 100 bar Temperature: 40 °C Static time: 150 min Dynamic time: 105 min Flow rates: 6 mL/min (static phase); 10:1 mL/min (dynamic phase)	[28]
	Bark	-	Pressure: 150 bar Temperature: 40 °C Flow rate: 1 L/min	[48]
*Bulnesia sarmientoi* Lorentz ex Griseb.	Bark	-	Pressure: 0–150 bar (20 min), 150–250 bar (20 min), 250–300 bar (10 min), 300–350 bar (10 min), 350 bar (2 h) Temperature: 40 °C	[110]
*P. sylvestris*	Bark	Ethanol	Pressure: 10.0 MPa (45 min), 20.0 MPa (45 min), 30.0 MPa (45 min) at 40 °C Temperature: 62.5 °C (45 min), 85 °C (45 min) at 30.0 MPa	[29]
*Cinnamomum cassia* (L.) J.Presl	Bark	-	Pressure: 600–650 bar Temperature: 45 °C	[58]
*Chamaecyparis obtusa* Endl.	Bark	-	Pressure: 350 bar Temperatures: 40 °C, 50 °C, 60 °C Flow rate: 145 mL/min	[71]
*Oplopanax horridus* Miq.	Root bark	-	Pressure: 360 bar Temperature: 40 °C Static time: 3 h	[72]
*Sclerocarya birrea* Hochst.	Stem bark	Ethanol	Pressure: 200 bar Temperature: 50 °C Flow rate: 23 g/min	[33]
*Ulmus davidiana* Planch.	Branch (with barks)	Ethanol	Pressure: 400 bar Temperature: 50 °C	[111]

**Table 5:** Advantages and disadvantages of extraction methods.

Extraction Method	Advantages/Disadvantages	Extraction Efficiency	Technical Complexity	Scale-Up Feasibility	Costs
Infusion/ Decoction	*Advantages*	- polar and heatstable phytochemicals	- simple equipment - easy to implement at lab scale	-	- low capital - safe operation (minimal safety/permit burden)
	*Disadvantages*	- selectivity (coextraction of nontarget compounds) - nonpolar compounds - tightly bound compounds - thermal degradation	-	- challenges for consistent mass transfer (uneven extraction in large beds)	- higher energy and downstream drying cost - time consuming
Maceration	*Advantages*	- polar compounds - gentle on thermolabile compounds - easy soluble compounds	- simple equipment - easy to implement at lab, pilot and simple industrial scales - low operator skill requirement	- scaleup is straightforward conceptually - applicable at lab, pilot and industrial scale	- low capital - safe operation (minimal safety/permit burden) while using ethanol or water
	*Disadvantages*	- low selectivity (coextraction of nontarget compounds) - low extraction efficiency	-	- challenges for consistent mass transfer (uneven extraction in large beds)	- time consuming process - high solvent consumption - higher energy and downstream drying cost
Soxhlet	*Advantages*	- high extraction completeness - good for broad polarity fractionation - reproducible and comparable	- simple, robust and wellunderstood - minimal operator training for lab/pilot work - straightforward protocols	- applicable at lab and pilot scale	-
	*Disadvantages*	- potential thermal degradation - poor selectivity (coextraction of nontarget compounds)	- more complex equipment required at industrial scale	- limited straightforward scaleup at industrial level	- time consuming process - high solvent and energy consumption
MAE	*Advantages*	- high efficiency and speed (especially for polar compounds) - better selectivity - reduced thermal degradation (optimized conditions) - easy to couple with other techniques	-	- viable scaleup paths on pilot and industrial scale	- lower time and energy consumption - lower solvent usage
	*Disadvantages*	- thermal degradation (without optimized conditions) - inconsistent extracts in poorly designed systems	- more complex lab equipment - more operator training needed for equipment handling	- challenging to maintain even mass/heat transfer at pilot and industrial scale	- higher costs for equipment - careful optimization needed for full potential
UAE	*Advantages*	- high efficiency - reduced thermal degradation - good for heatsensitive compounds - solvent flexibility	- relatively low complexity at lab and pilot scales	- viable scaleup paths on pilot and industrial scale	- lower time and energy consumption - lower solvent usage
	*Disadvantages*	- higher dependence on matrix particle size - limited selectivity for nonpolar fractions	-	- challenges in controlling cavitation field at industrial scale to avoid low extraction zones	- higher costs for equipment at industrial scale
SFE	*Advantages*	- high selectivity for lipophilic compounds - polarity extension via cosolvent addition - multistage fractionation capability - good extract quality - solvent free final extracts	-	-	- CO_2_ recyclability benefits costs at lab scale - no downstream costs needed (solvent free extarcts)
	*Disadvantages*	- poor selectivity for polar compounds without cosolvents	- high equipment complexity - highly trained operators needed	- careful design needed for scaleup - pilot scale validation required	- high equipment costs at all scales - intensive optimization processes required - high energy and solvent (CO_2_) consumption at pilot and industrial scales
Ionic liquids	*Advantages*	- access to compounds not recovered by conventional solvents - tunable selectivity by solvent choice - compatibility with other methods (MAE/UAE)	-	- feasible at lab scale by combining with UAE/MAE	-
	*Disadvantages*	- compound recovery difficulty results in lower yields - masstransfer limits at high concentrations due to high viscosity	- more complex downstream processing of extracts	- more complex for industrial scaleup compare to simple MAE/UAE	- time and energy consumption on downstream processing - recurring ionic liquids cost
Enzymatic	*Advantages*	- increased accessibility of bound phenolics, tannins and glycosides - increased recovery of specific phytochemicals - reduces thermal and oxidative degradation (neutral pH, moderate temperatures) - compatibility with other methods (MAE/UAE)	-	- scaleup is possible, but needs careful engineering	-
	*Disadvantages*	- variable effectiveness by matrix and target compounds - potential for product modification during extraction	-	- maintaining constant parameters challenging at large scales	- time consuming because of incubation time and well prepared designs - enzyme preparations increase costs
Deep eutectic solvent	*Advantages*	-			
	*Disadvantages*				

**Table 6:** Yields and phytochemical profiles of extracts obtained with different methods.

Species	Extraction Method	Phytochemical Profile	References
*Picea* spp.	UAE	Sinapic acid, Vanilic acid, Quercetin, Taxifolin	[126]
	Decoction/Infusion		
	Maceration	Astringin, Piceid, Isorhapontin, Pieaceatannol, Resveratrol, Isorhapontigenin	[127]
	SFE combined with UAE/MAE/Maceration	Abietic acid, Dehydroabietic acid, methyl abietate, Piceasides (A-H), Isorhapontin, Piceid, Astringin, Flavonoids (e.g., Taxifolin, Quercetin, Isorhamnetin, Catechin, Quercetin), Ferulic acid, Quinic acid	[48]
	UAE	Catechin, Epicatechin, α-pinene, β-pinene, Camphene, 3-carene, α-Phellandrene, Limonene, Sabinene, Myrcene, Tricyclene	[52]
	MAE		
	DES	Polyphenols	[128]
*Pinus* spp.	MAE	Quinic acid, p-Hydroxy benzoic acid hexoside, Sucrose, Taxifolin, Catechin/Epicatechin, Proanthocyanidins	[95]
	SFE with EtOH co-solvent	Unsaturated fatty acids, Sterol esters, Resin acids, Abietic acid, Dehydroabietic acid	[29]
	Maceration	Caffeic, Ferulic, Cinnamic, chlorogenic, Vanillic, Dihidroxybenzoic, Ellagic acids, Catechin/Epicatechin, Gallocatechin, Naringenin, Quercetin, Apigenin, Taxifolin, Resveratrol	[39]
	UAE	Catechin, Taxifolin	[34]
	MAE		
*Quercus* spp.	UAE	Polyphenols, Tannins, Gallic acid, Catechin, Taxifolin, Vanillic acid, Caffeic acid	[1,30]
	MAE		
	Maceration	Flavonoids, Hydroxycinnamic acids, Proanthocyanidins	[27]
	Decoction		
	UAE	Gallic, Caffeic, Ellagic, Protocatechuic, Vanillic acids, Catechin/Epicatechin, Epigallocatechin	[129]
	Soxhlet	Friedelin	[130]
	SFE with EtOH co-solvent		
	DES	Fatty acids, Small alcohols and acids, Aromatic compounds, Sugars, Terpenoids	[131]
*Salix* spp.	UAE	Salicin, Chlorogenic acid, Gallic acid, p-Hydroxybenzoic acid, Syringic acid, p-Coumaric acid, trans-Cinnamic acid, Epicatechin, Rutin, Quercetin, Naringenin	[14]
	Maceration	Piceol, Picein, Catechin, Eriodictyol Naringenin, Salicylic acid, Glicosides of Quercetin, Naringenin, Eriodictyol and Procyanidins	[12]
	SFE	Salicin, Saligenin, Salicortin, Catechin, Quercetin, Naringenin, Ferulic, Sinapic, p-Coumaric, Syringic, Protocatechuic, p-Hydroxybenzoic, Caffeic acids.	[63]
	MAE	Phenolics, Flavonoids, Tannins	[8]
	DES	Gallic acid, Chlorogenic acid, Vanillic acid, Syringic acid, p-Coumaric acid, Sinapic acid, Cinnamic acid, Epicatechin, Rutin, Quercetin, Naringenin	[132]

## Abbreviations

The following abbreviations are used in this manuscript:

MAE	Microwave-assisted extraction
UAE	Ultrasound-assisted extraction
SFE	Supercritical fluid extraction
DES	Deep eutectic solvent
HBA	Hydrogen bond acceptor
HBD	Hydrogen bond donor
SC-CO_2_	supercritical carbon dioxide
MeOH	Methanol
EtOH	Ethanol
NaOH	Sodium hydroxide
